# Integrated volatile metabolome and transcriptome analyses provide insights into the warm aroma formation elicited by methyl jasmonate in carrot root

**DOI:** 10.3389/fpls.2024.1467957

**Published:** 2024-09-18

**Authors:** Guang-Long Wang, Jia-Qi Wu, Yang-Yang Chen, Yu-Jie Xu, Ya-Hong An, Xu-Qin Ren, Ai-Sheng Xiong

**Affiliations:** ^1^ School of Life Science and Food Engineering, Huaiyin Institute of Technology, Huaian, China; ^2^ Jiangsu Provincial Agricultural Green and Low Carbon Production Technology Engineering Research Center, Huaiyin Institute of Technology, Huaian, China; ^3^ State Key Laboratory of Crop Genetics & Germplasm Enhancement and Utilization, College of Horticulture, Nanjing Agricultural University, Nanjing, China

**Keywords:** volatile metabolome, transcriptome, MeJA, terpenes, transcription-structural genes, carrot

## Abstract

Carrot is a highly significant vegetable cultivated worldwide and possesses a unique aroma with abundant edible and medicinal values. However, it remains largely unknown whether jasmonic acid could regulate aroma formation in carrot. Here, an integrated analysis of the volatile metabolome and transcriptome of carrot roots exposed to different concentrations of methyl jasmonate (MeJA) was performed. The results revealed 1,227 volatile organic compounds and 972 differential accumulated metabolites, with terpenes representing the largest portion. MeJA treatment evidently increased the relative odor activity values as well as the accumulation of most volatile compounds. In addition, 4,787 differentially expressed genes were identified and subjected to function enrichment analysis, indicating a role of terpene biosynthesis and metabolism in response to MeJA application. A network consisting of 4,680 transcription factor-structural pairs that showed highly significant positive correlations was constructed, which may be utilized as genetic targets for examining terpene accumulation and aroma formation elicited by methyl jasmonate. The results from the present work substantially improved our understanding of MeJA-mediated aroma formation in carrot.

## Introduction

Plant volatile organic compounds (VOCs) are a class of volatile products generated during metabolic processes, mainly consisting of terpenoids, alcohols, aldehydes, ketones, esters, phenols, and organic acids (Abbas et al., 2022; Brosset and Blande, 2022). The composition and proportion of these compounds contribute to a special aroma of each plant species. The types and contents of plant VOCs vary in response to different metabolic processes and enzyme catalysis, resulting in different VOC characteristics in different plants or the same plant at different physiological states or developmental stages (Bao et al., 2023; Dady et al., 2023). They play an important role in attracting pollinators and seed spreaders, resisting against biotic and abiotic attack, as well as in signal transduction between plants (Hu, 2022; Kessler et al., 2023; Ninkovic et al., 2021). Therefore, studying plant VOCs can not only help to understand plant physiology and metabolism characteristics, but also provide theoretical basis and practical guidance for biological control of plant diseases and pests, and promote innovation and development of plant germplasm resources with special aroma.

Unlike the aboveground parts, there is still a lack of research on the types and regulation of volatile metabolites in plant roots. However, for many root vegetables, volatile metabolites determine the quality of the product and affect people’s preference and ways of consumption to some extent. In radish, sulfur and nitrogen-containing compounds were deemed to play an important role in flavor formation, with diallyl sulfide and dimethyl disulfide being the primary sulfides responsible for the unpleasant flavor in radish (Gamba et al., 2021; Cai et al., 2024). In fresh turnip, isothiocyanato-cyclopropane and (2-isothiocyanatoethyl)-benzene were identified as the predominant volatiles (Xue et al., 2020). These studies have increased our understanding of volatile compounds in root vegetables, but the accumulation patterns and regulatory mechanisms of these substances under specific conditions in root vegetables are still unclear.

Carrots are a nutritious root vegetable belonging to the family Apiaceae, which are annual or biennial herbaceous plants. It is abundant in various nutrients such as carotene, ascorbate, and dietary fibre, and has various health benefits such as protecting vision, enhancing immunity, and promoting digestion and absorption of human bodies (Deng et al., 2023; Duan et al., 2024; Loarca et al., 2024). Whether eaten raw, cooked, or juiced, carrots can bring health and deliciousness to people. Therefore, carrots play an important role in daily diet and are deeply loved by people. Carrots not only have high nutritional value, but also have a pleasant flavor (Muchlinski et al., 2020; Yahyaa et al., 2018). The aroma of carrots comes from the volatile compounds within carrot plants, which give them a unique aroma. This odor may vary slightly due to factors such as carrot variety, tissues, growth environment, and maturity (Fukuda et al., 2013). These compounds mainly consist of α-pinene, β-myrcene, limonene, (E)-β-caryophyllene, γ-terpinene, bornyl acetate, and cymene, most of which belong to the terpenoids (Quarrell et al., 2023; Tian et al., 2024). It is reported that terpenoids account for nearly 98% of the VOCs in carrot tissues.

Jasmonic acid (JA) is an endogenous growth regulator present in higher plants and is a derivative of fatty acids (Li et al., 2022). It is widely present in plant tissues and organs such as flowers, stems, leaves, and roots, and plays an important role during plant growth and development processes (Li et al., 2021; Nguyen et al., 2022). JA is involved in root elongation, flower development, leaf senescence, stomatal opening and closing, epidermal hair initiation (Chang et al., 2024; Furuta et al., 2024; Han et al., 2023b). In addition, JA can mediate plant resistance against insects and pathogens and regulate plant response to stress such as drought, high temperature, ozone, and ultraviolet radiation (Escobar-Bravo et al., 2019; Raza et al., 2021; Zhao et al., 2024). Further studies have shown that JA can also induce the accumulation of secondary metabolites, such as anthocyanins and flavonoids, which in turn contribute to the formation of crop quality (Fang et al., 2023; Wang et al., 2022).

Although the roles of JA in VOC accumulation have been discovered in some plant species, whether JA could induce enhanced aroma formation in carrot is still unclear. Here, we evaluated the dynamics of VOC accumulation in carrot plants exposed to exogenous methyl jasmonate (MeJA). In addition, we focused on the changes of terpenoids, the characteristic volatile substances of carrots, during this process, and analyzed the structural genes and regulatory factors that regulate the synthesis of terpenoids. We constructed a possible regulatory network of MeJA-mediated terpenoid accumulation in carrots. The results of the current study may lay foundation for the research on JA-regulated volatile accumulation and offer new genetic resources and targets for quality regulation in crops.

## Materials and methods

### Plant materials and experimental design

‘Kurodagosun’ is a carrot variety with high yield and quality, and is widely cultivated in Asia. It has an orange color on the root surface with a dense texture in the flesh. In this study, the seeds of carrot variety ‘Kurodagosun’ were sown in pots holding a mixture of vermiculite and organic matter, and were transferred to a greenhouse for seedling rearing. After 38 d, each pot was sprayed with 300 mL of 100 μM MeJA (MeJA100), 200 μM MeJA (MeJA200), and 10 mM sodium diethyldithiocarbamate trihydrate (DIECA, a JA biosynthesis inhibitor) weekly via root irrigation, with clean water (CK) as the control. Carrot plants were totally exposed to different concentration of MeJA five times, and the carrot fleshy root samples from different treatments were respectively collected at 70 days after sowing. The harvested samples were immediately frozen in liquid nitrogen and stored in an ultra-low temperature freezer for further analysis.

### Sample preparation and GC-MS analysis of VOCs

The collected carrot root samples were ground into a fine powder in liquid nitrogen. Approximately 0.5 g of the powder was immediately transferred to a 20 mL head-space vial (Agilent, Palo Alto, CA, USA) harboring a saturated NaCl solution to inhibit enzyme reaction. The crimp-top caps equipped with TFE-silicone headspace septa (Agilent) were introduced to tightly seal the vials. For SPME analysis, each vial was placed in a 60°C environment and vibrated for 5 min, followed by headspace extraction for 15 min at 60°C with insertion of an Agilent 120 µm DVB/CWR/PDMS fibre (CA, USA). Desorption of the VOCs from the fibre coating was implemented at 250°C for 5 min in the splitless mode.

The volatile metabolites were identified and determined by an Agilent Model 8890 GC and a 7000D mass spectrometer (Agilent), equipped with a 30 m × 0.25 mm × 0.25 μm DB-5MS (5% phenyl-polymethylsiloxane) capillary column. High-purity helium was utilized as the carrier gas at a linear velocity of 1.2 mL/min, with the temperature of the injector maintained at 250°C. The temperature gradient of the oven was set as described by a previous report (Wang et al., 2021). Mass spectra were noted in the electron impact ionization mode at 70 eV. The temperatures of quadrupole mass detector, ion source, and transfer line were adjusted to 150, 230, and 280°C, respectively. The selected ion monitoring (SIM) mode was applied for the precise identification and quantification of analytes. Volatile organic compounds were differentiated by comparing the mass spectra with the data system library (MWGC or NIST) and linear retention index. The average values of each metabolite in different treatment groups were generated from six biological samples (**Supplementary Table 1**). Compounds with a fold change ≥ 2 or ≤ 0.5 in contents and variable importance in project (VIP) > 1 were referred to differential metabolites (**Supplementary Table 2**). The principal component analysis (PCA) and K-Means clustering were carried out by R base package with data scaled by unit variance.

### Transcriptome sequencing

Transcriptome sequencing data of each treatment group were produced from three biological replicates. Total RNA from carrot root samples was isolated by ethanol precipitation and CTAB-PBIOZOL and dissolved with 50 µL of DEPC-treated water. Subsequently, a Qubit fluorescence quantifier and a Qsep400 high-throughput biofragment analyzer were adopted to identify and quantify RNA concentration and integrity. To construct a cDNA library, mRNAs with polyA tails were enriched by Oligo(dT) magnetic beads, since most eukaryotic mRNAs harbor a polyA tail. Subsequently, a fragmentation buffer was added to break the RNA into short fragments, which was then used as a template to synthesize the first strand cDNA with random hexamer primers. Then, buffer solution, dNTPs, and DNA polymerase were added to yield the second strand cDNA. The purified second strand cDNA was subjected to end repair, dA-Tailing, and adapter ligation. Then, DNA magnetic bead purification, fragment selection, and ligated product PCR amplification were carried out to obtain the final cDNA library. After library inspection, different libraries were pooled according to the target output data volume and sequenced using the Illumina NovaSeq 6000 sequencing platform by Metware Biotechnology Co., Ltd. (Wuhan, China). The generated raw reads were uploaded in the NCBI Sequence Read Archive (SRA) database with a Bioproject accession number PRJNA1150744.

### Differentially expressed genes identification

The raw data were filtered by removing reads below the quality cutoff. Then the generated clean data were mapped to the reference genome (https://ftp.ensemblgenomes.ebi.ac.uk/pub/plants/release-55/fasta/daucus_carota/dna) using HISAT2 software. The genes with an absolute value of log2 fold change ≥ 1 were recognized as differentially expressed genes (DEGs). The DEGs were then subjected to functional annotation and enrichment analysis, such as k-means, GO enrichment. The gene co-expression network (P ≤ 0.01, r ≥ 0.9) between transcription factors (TFs) and terpenoid synthase genes was visualized by using Cytoscape (v.3.9.1) software.

### Quantitative real-time PCR validation

To determine the accuracy of the expression trends of candidate genes from RNA-seq, quantitative real-time PCR (qRT-PCR) was introduced. In brief, the synthesis of cDNA was strictly carried out according to the instructions of the HiScript II QRT SuperMix for qPCR (Nanjing Novozymes Biotechnology Co., Ltd.) reverse transcription kit. The primers designed for qRT-PCR were displayed in **Supplementary Table 3**. The reactions were performed on a CFX96 fluorescence quantitative PCR instrument (Bio Rad, USA) using carrot *DcACTIN* as an internal reference gene (Tian et al., 2015). The specific operation programmes strictly conformed to the Cham Q SYBR qPCR Master Mix kit and its instructions provided by Nanjing Novozymes Biotechnology Co., Ltd. The relative expression levels of the target genes in different samples were determined using the 2^-ΔΔCT^ method (Livak and Schmittgen, 2001).

## Results

### Metabolite analysis in the MeJA-treated root samples

**Figure 1:**
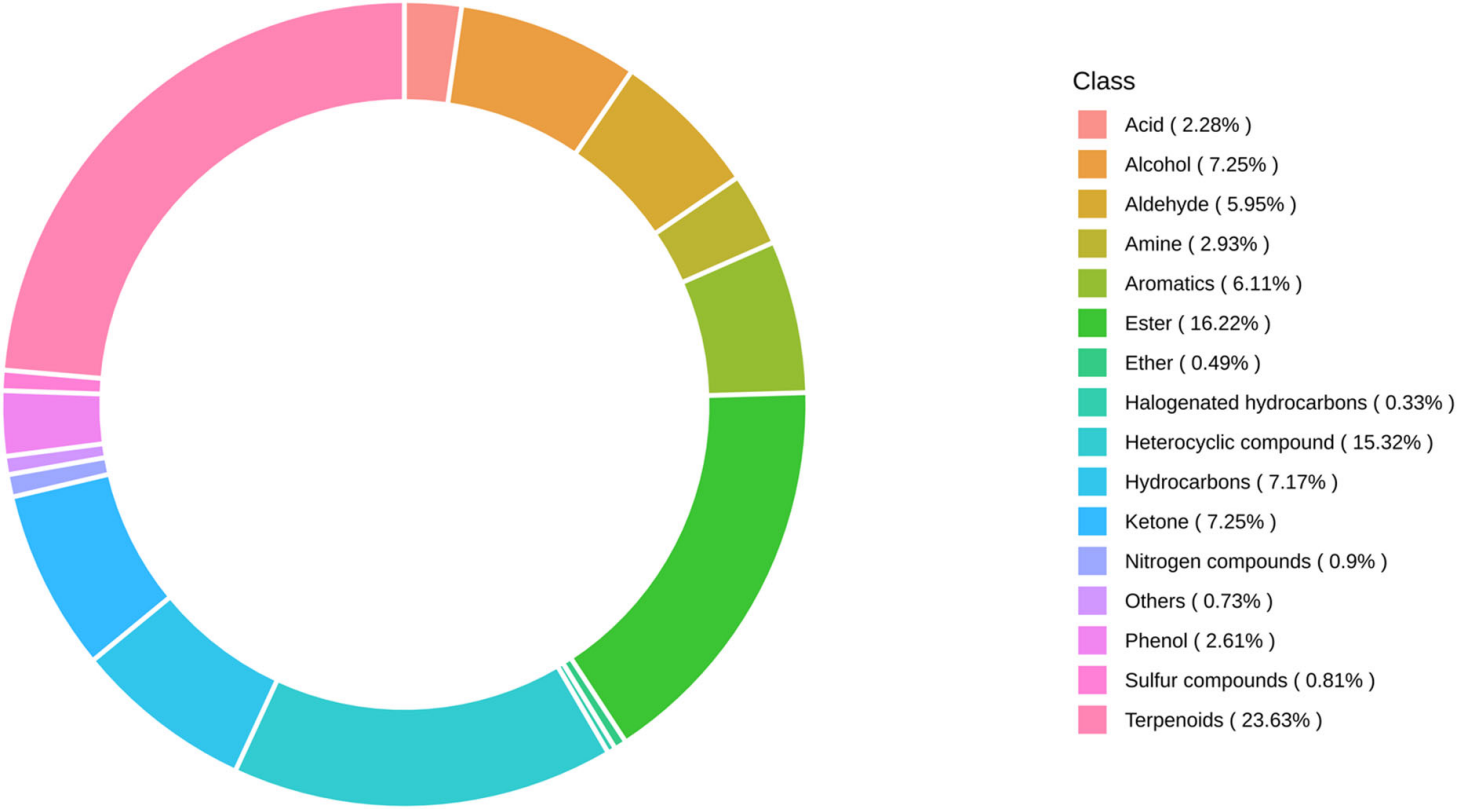
Distribution of volatile organic compounds in carrot root samples exposed to MeJA.

**Figure 2:**
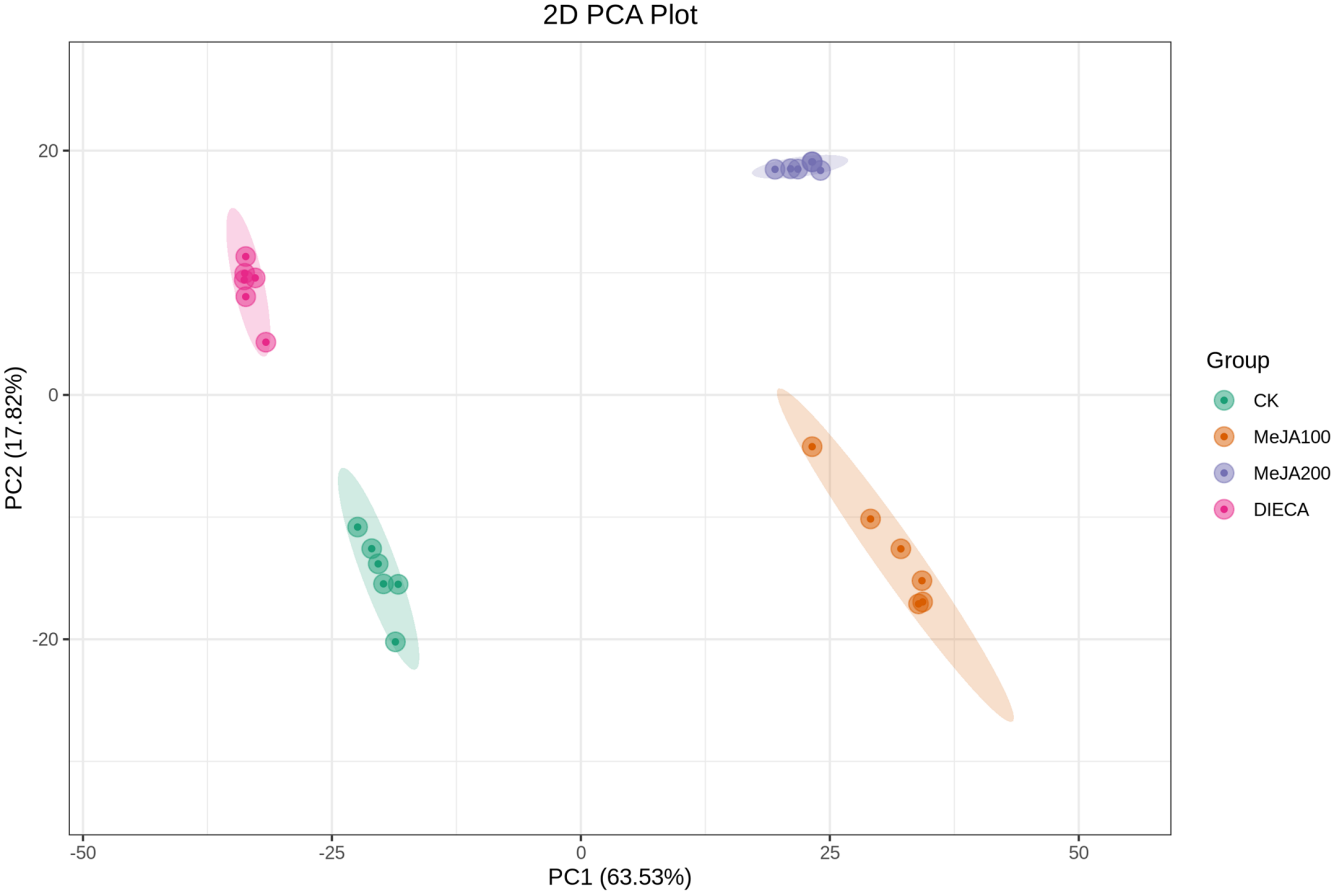
PCA analysis of the MeJA-treated samples. PC1 and PC2 represented the first and second principal component, respectively, whereas percentages indicated the explanatory power of these principal components on the dataset. Each point in the figure stood for a biological sample, and samples from the same group were represented by the same color.

**Figure 3:**
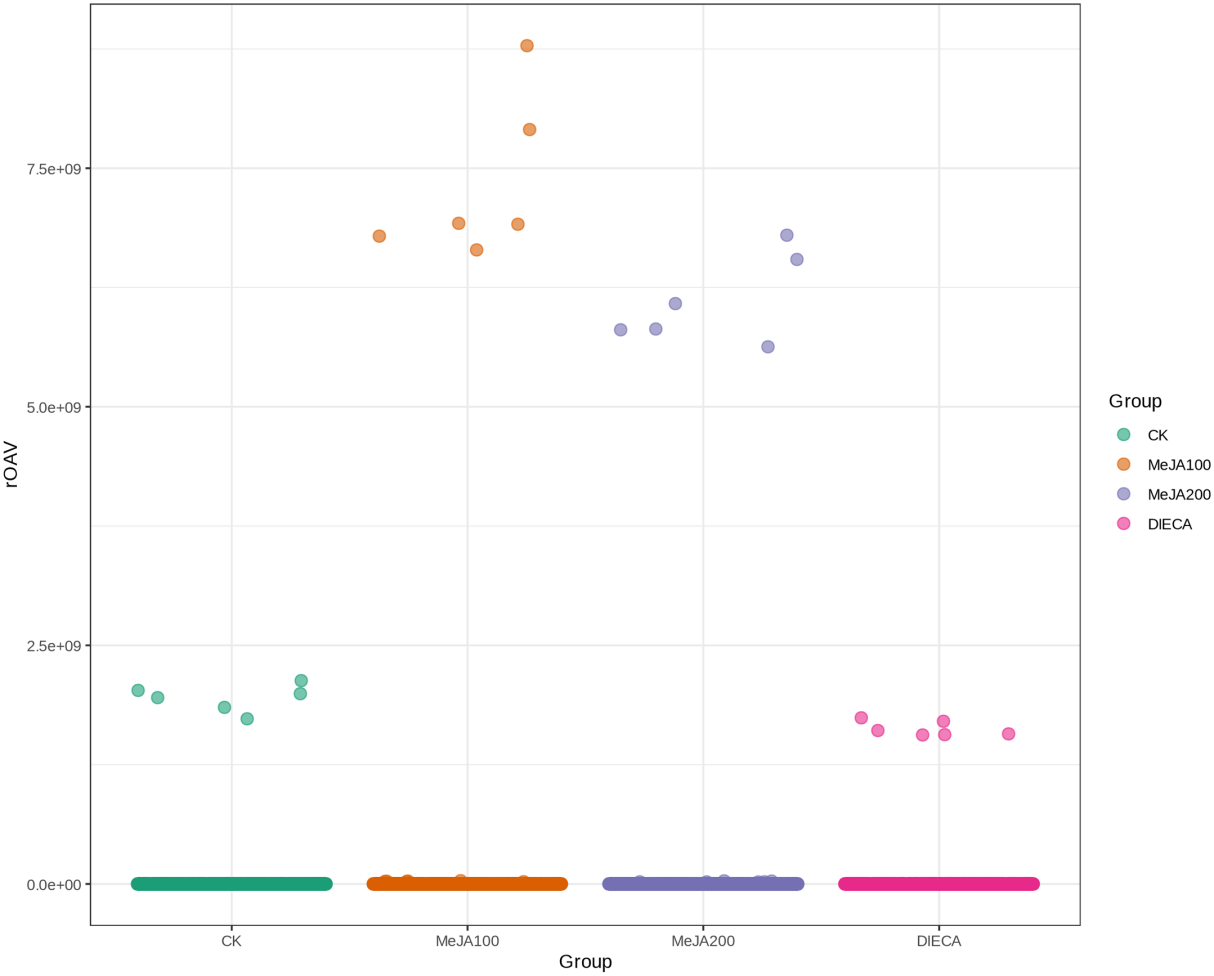
Scatter plot of relative odor activity values for MeJA-treated root samples. The horizontal axis represented different groups, whereas the vertical axis indicated the rOAVs of flavor compounds.

A total of 1227 VOCs were identified in 12 samples (four groups with three biological replicates). In terms of their chemical structures, these VOCs can be divided into 16 groups (**Figure 1**). Among them, terpenoids accounted for the largest group, followed by esters, heterocyclic compounds, alcohols, and ketones. It is convenient to gain a preliminary understanding of the overall metabolite differences across all groups and the variability magnitude among samples within the same group by conducting principal component analysis (PCA). The PCA results, showing the trend of metabolome separation among groups, indicated there were no evident differences detected in metabolome within the sample groups (**Figure 2**). Compared with the control group, the relative odor activity value (rOAV) was obviously higher in MeJA-treated carrots, whereas a slight a decrease was observed in root samples exposed to DIECA (**Figure 3**).

### Characterization and function analysis of differential metabolites

**Figure 4:**
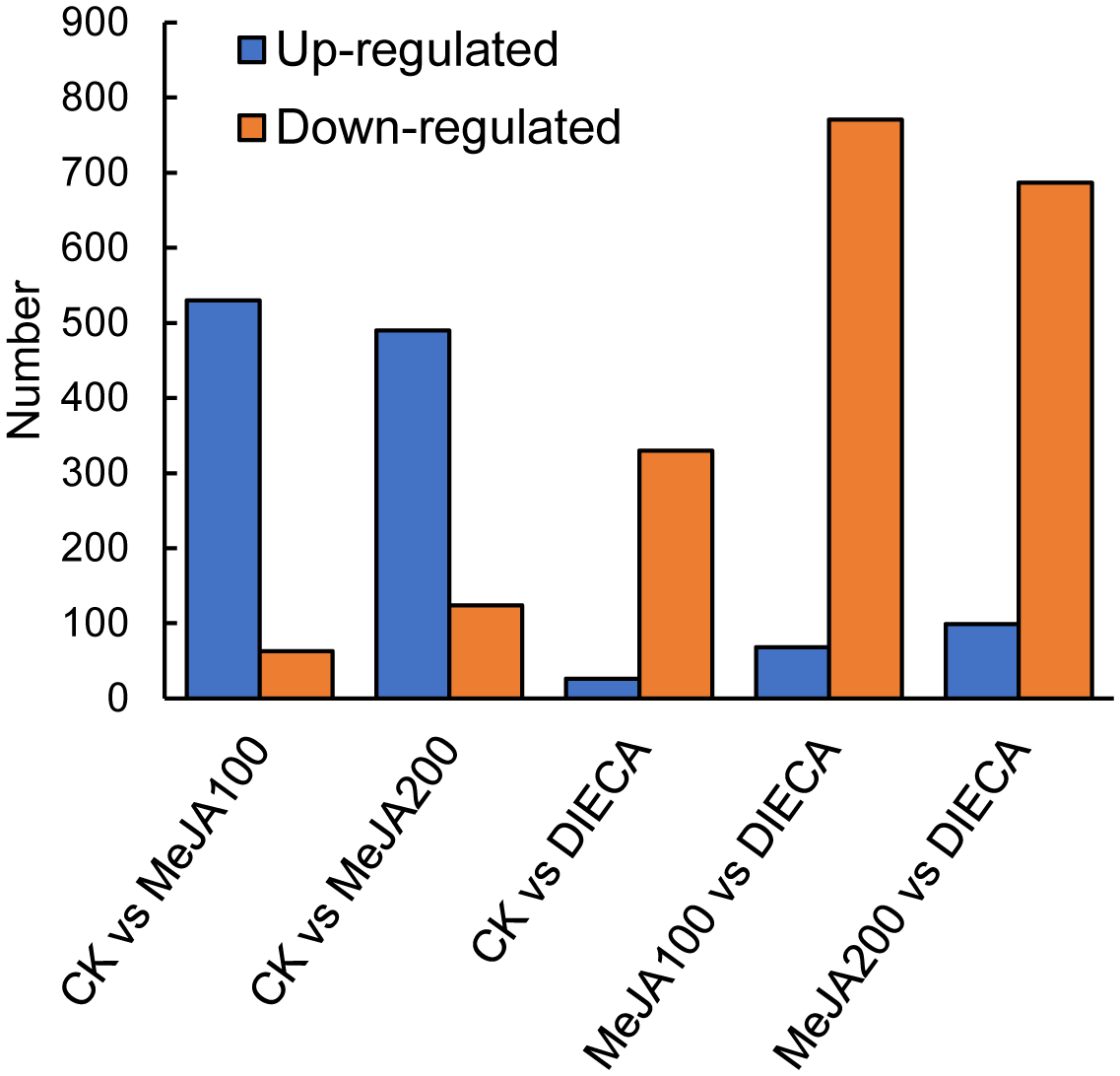
Comparison of differential accumulated metabolites between MeJA-treated groups.

**Figure 5:**
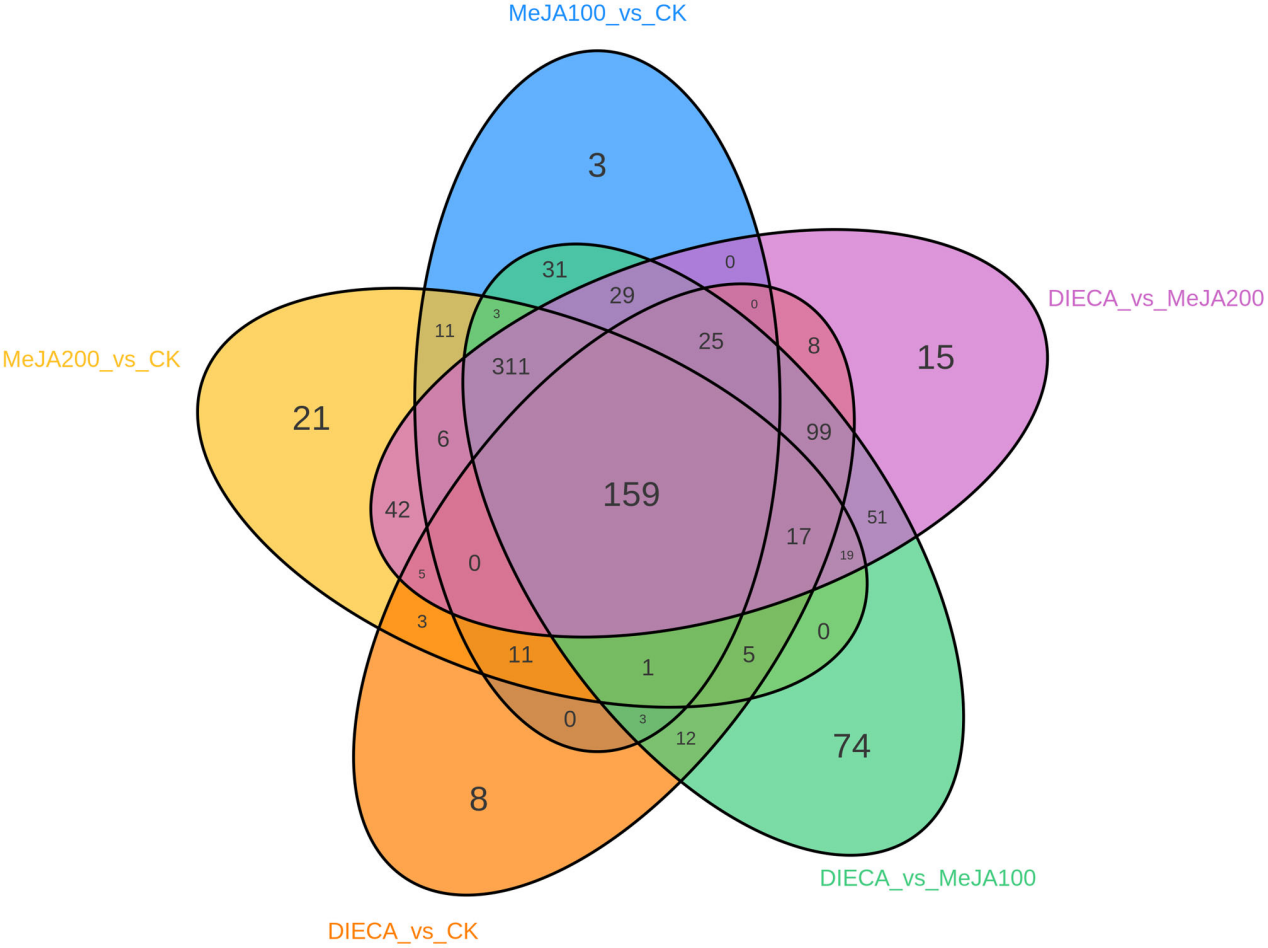
Venn diagram of distribution of differential metabolites in five comparisons.

**Figure 6:**
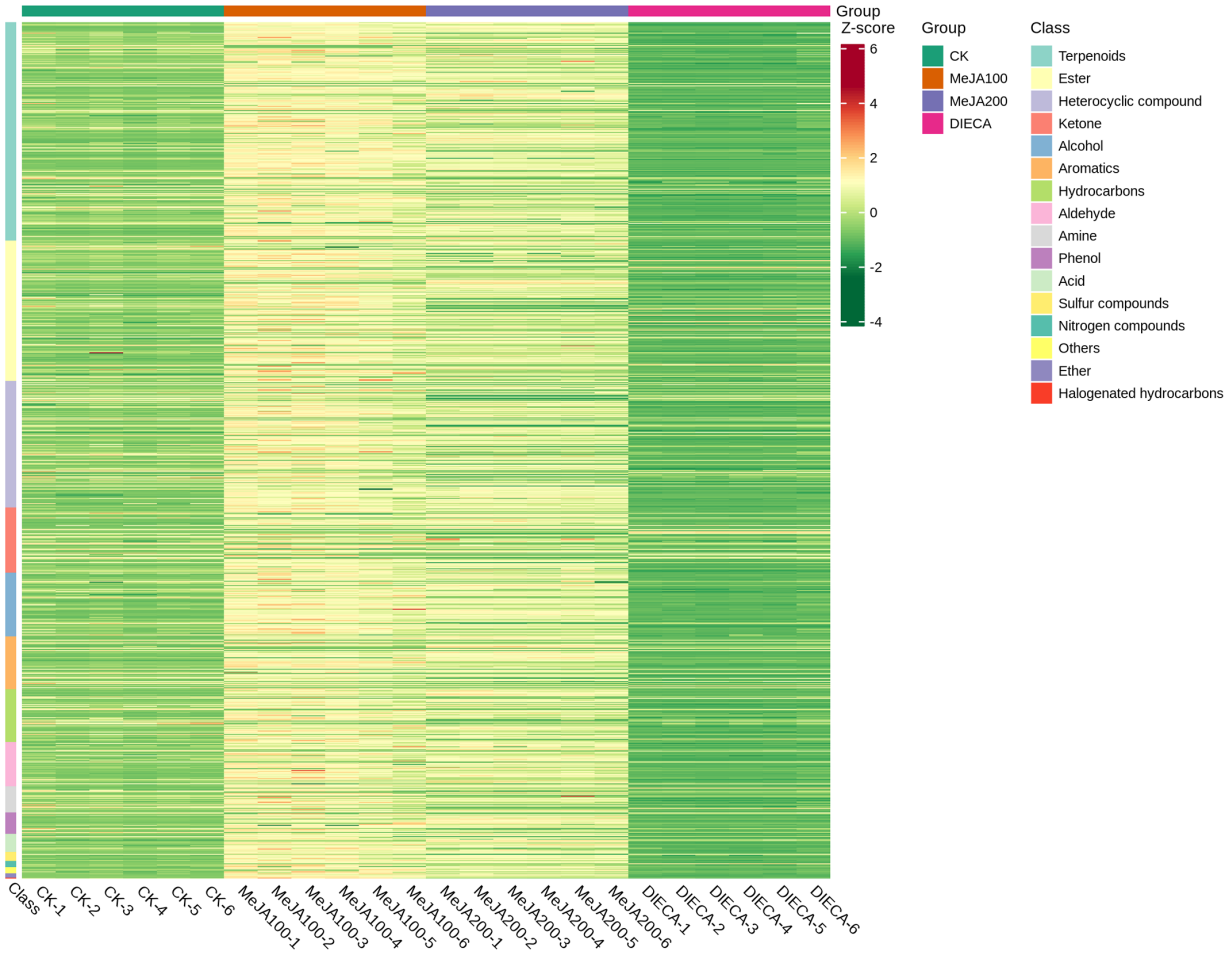
Heat map of the differential volatile metabolites in MeJA-treated carrot roots. The red and green represented high and low levels of metabolites, respectively.

**Figure 7:**
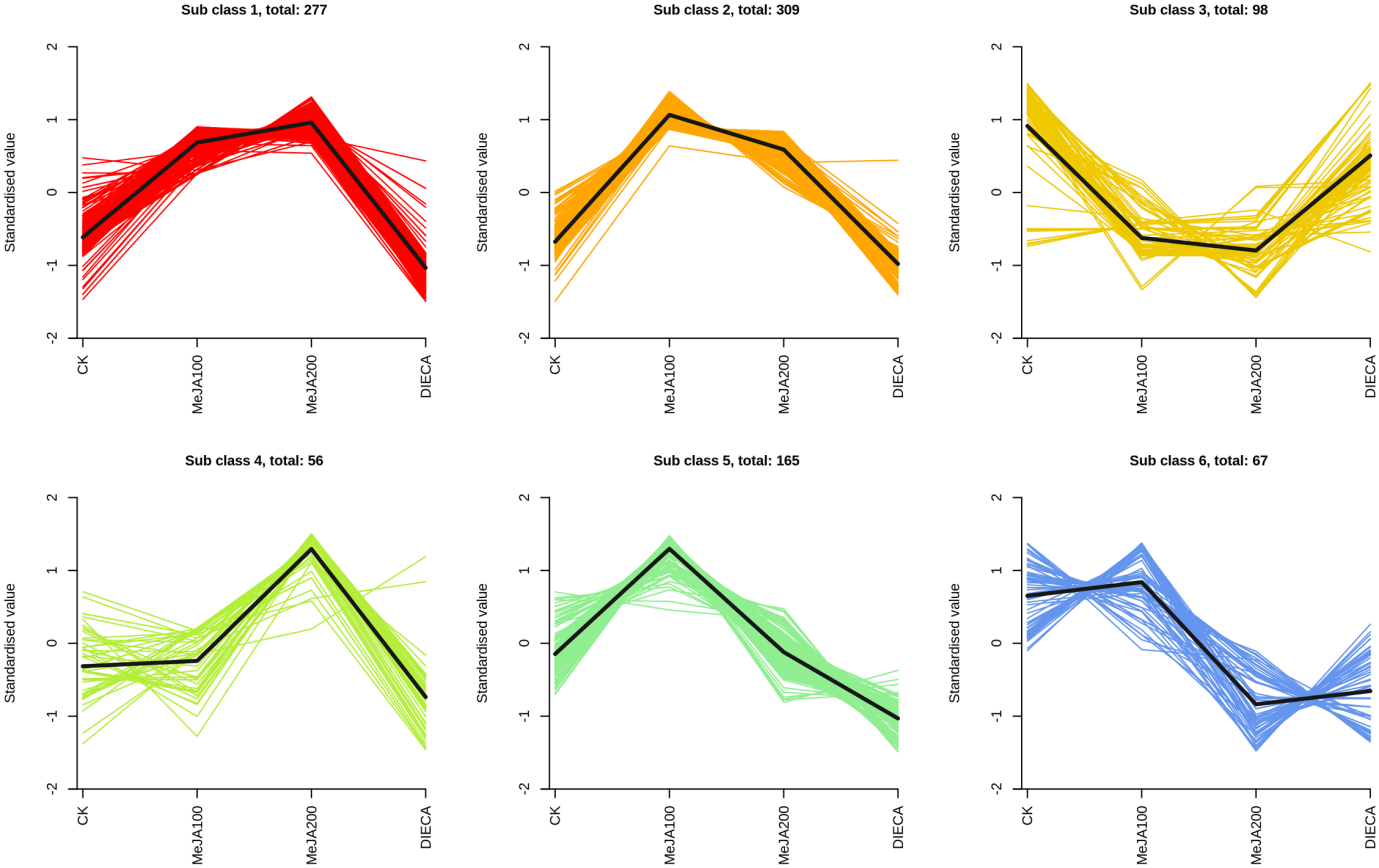
Six K-means clusters showed differential accumulated trends of volatile metabolites.

To characterize the dynamic changes of metabolites in response to MeJA application, comparison of the volatile metabolites among the four samples (CK, MeJA100, MeJA200, and DIECA) was carried out (**Figure 4**). There were 530, 490, 26, 68, and 99 up-regulated and 63, 124, 330, 771, and 687 down-2regulated volatile compounds detected in “CK vs MeJA100”, “CK vs MeJA200”, “CK vs DIECA”, “MeJA100 vs DIECA” and “MeJA200 vs DIECA” comparisons. Obviously, Exogenous MeJA application substantially increased the accumulation of volatile metabolites, whereas DIECA did the opposite. Totally, 972 differential metabolites were determined among the five comparisons, accounting for 79.22% of all the volatile compounds detected (**Figure 5**). Among them, 159 metabolites differed in concentrations across all the comparisons. Then, the differentially accumulated metabolites were then visualized by a heat map (**Figure 6**). The figure showed that the concentrations of most of the differential metabolites were the highest in MeJA-treated groups, whereas the lowest levels were detected in the plants exposed to DIECA, an inhibitor for JA biosynthesis. To further excavate the general accumulation trends of VOCs in response to MeJA treatment, the K-means clustering algorithm was introduced to classify the different characteristics of the accumulation profiles of differential metabolites. Overall, six different accumulation classes (Class 1 to Class 6) were identified, with 277, 309, 98, 56, 165, and 67 members, respectively (**Figure 7**).

### Identification of the differentially expressed genes during MeJA treatment

**Figure 8:**
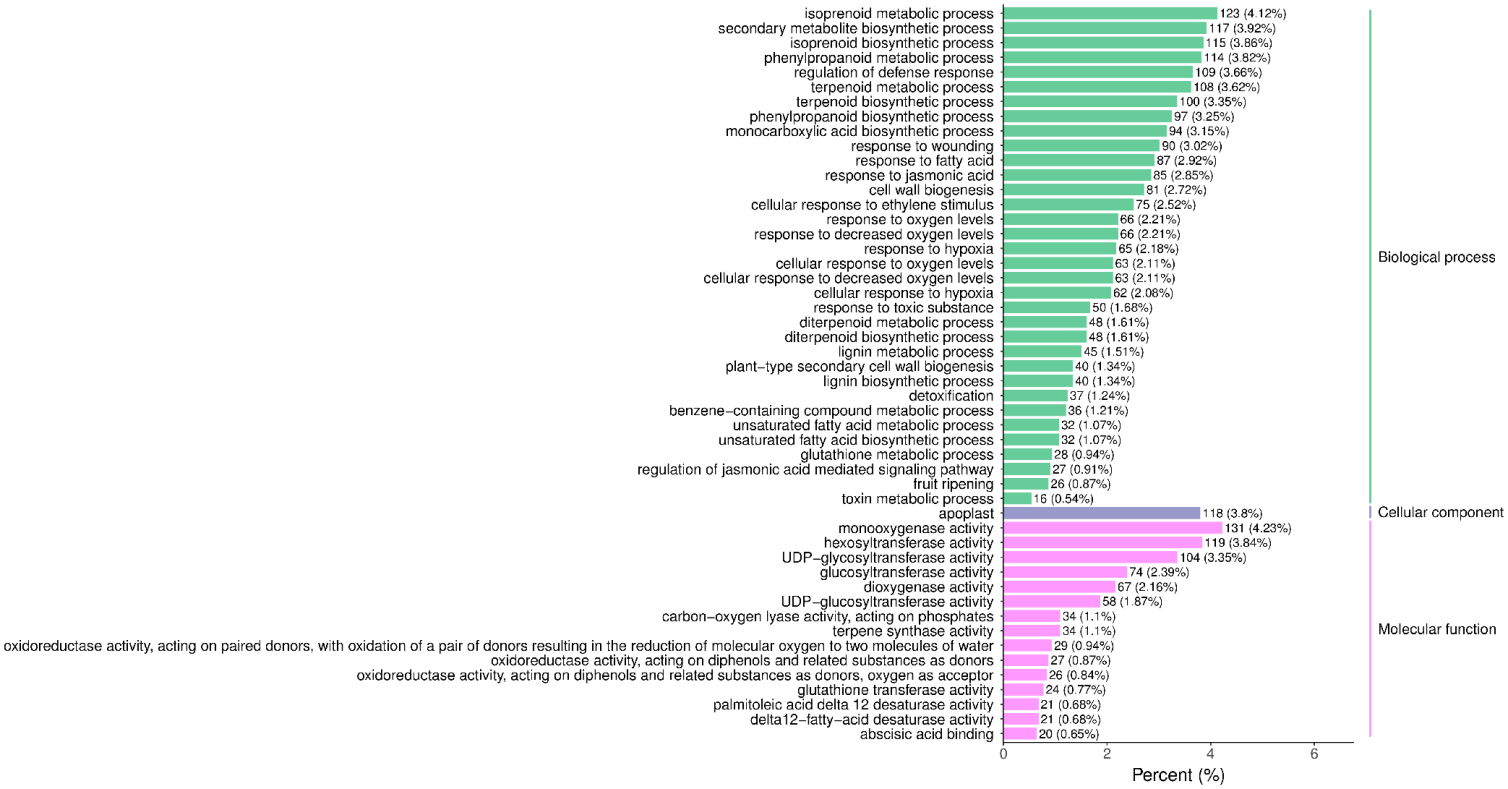
GO enrichment analysis of the differentially expressed genes. The top 50 GO terms with lowest p-value were displayed.

**Figure 9:**
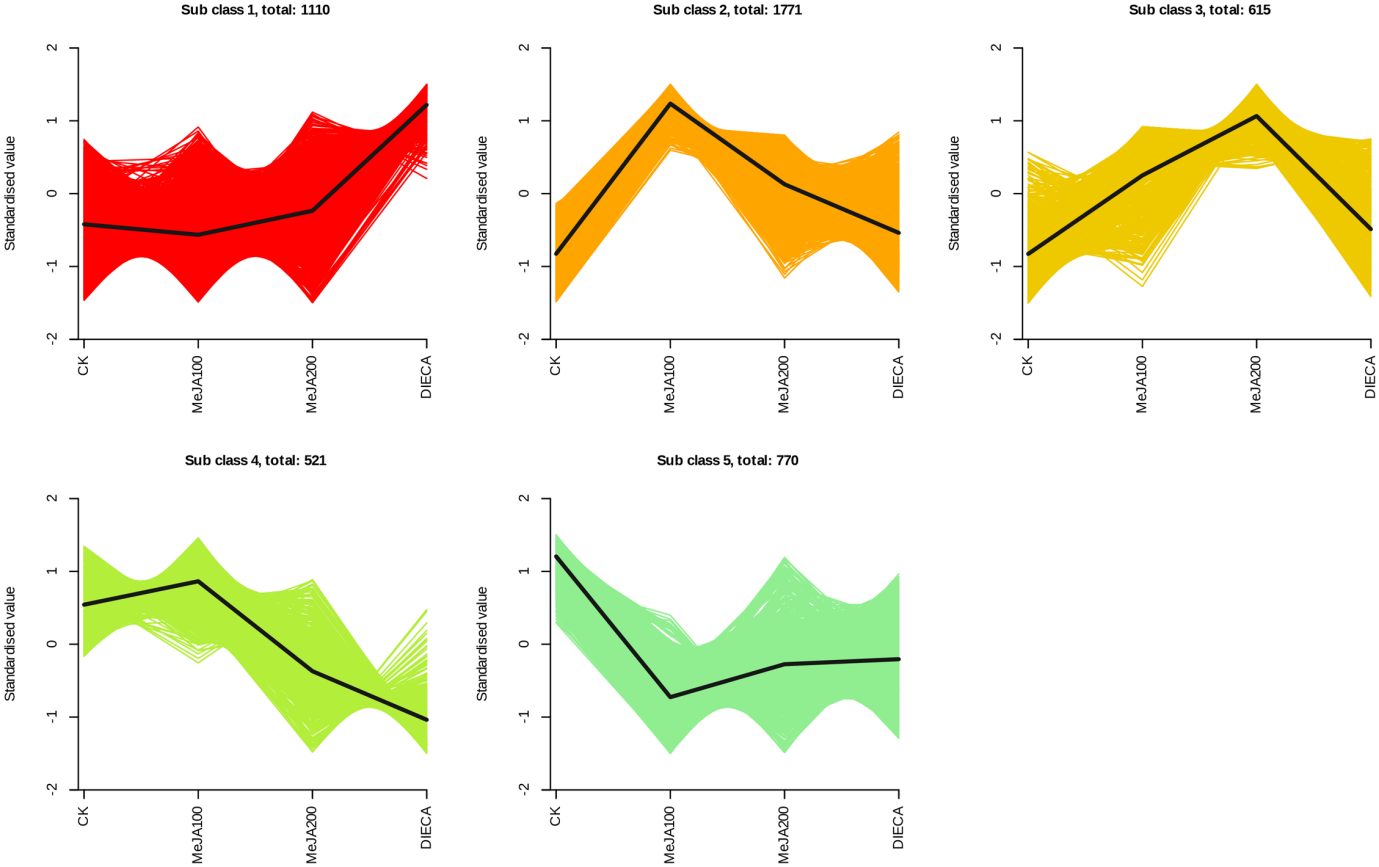
Different expression patterns of differentially expressed genes identified by K-means analysis.

The raw reads were filtered and mapped to the carrot reference genome (**Supplementary Table 4**). Approximately 90% of the clean reads of each sample were respectively mapped to the reference genome. Specifically, 2004, 1360, 943, 815, and 476 up-regulated and 664, 480, 678, 1829, and 1109 down-regulated genes were respectively determined in “CK vs MeJA100”, “CK vs MeJA200”, “CK vs DIECA”, “MeJA100 vs DIECA” and “MeJA200 vs DIECA” comparisons. A total of 4,787 differentially expressed genes were identified in the various groups. The DEGs detected were then subjected to Gene Ontology (GO) enrichment analysis (**Figure 8**). In the biological process group, “isoprenoid metabolic process”, “secondary metabolite biosynthetic process”, and “isoprenoid biosynthetic process” were the most enriched terms, followed by “phenylpropanoid metabolic process”, “regulation of defense response”, “terpenoid metabolic process”, and “terpenoid biosynthetic process”. In the molecular function cluster, “monooxygenase activity” and “hexosyltransferase activity” were the most abundant terms. In order to further understand the expression profiles of DEGs among the MeJA treatments, k-means were utilized and the DEGs were apparently classified into 5 subcategories (**Figure 9**). Given the relative odor activity values and the metabolite accumulation patterns, the 2,386 DEGs from class 2 and 3 were further analyzed.

### Analysis of the terpenoid accumulation and terpenoid synthase expression

**Figure 10:**
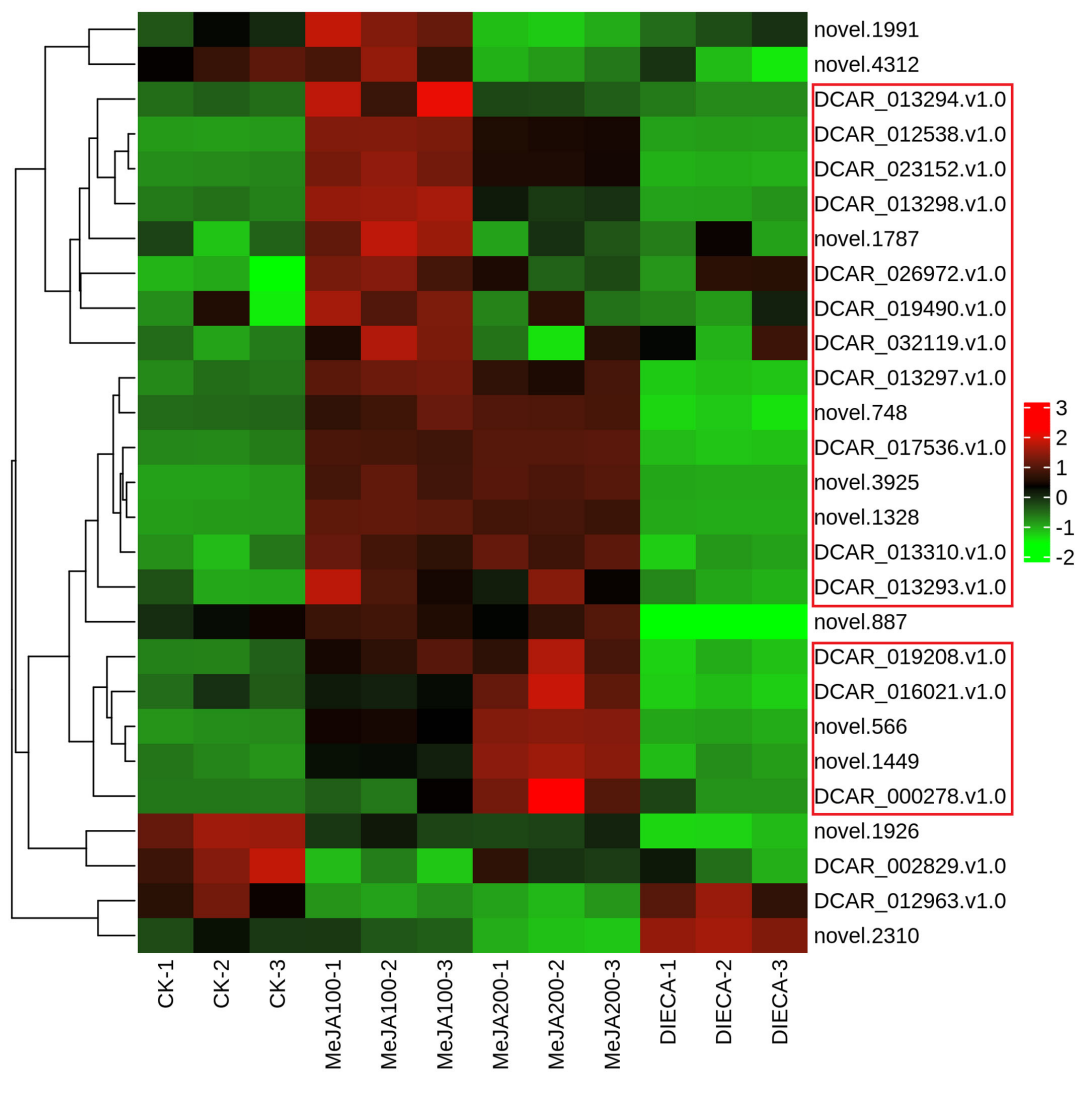
Heat map representing the expression of terpenoid synthase genes in different samples after MeJA application. The red and green indicated high and low expression, respectively.

It is reported that the specific aroma in carrot derived mainly from the terpenoids within carrot tissues. Under normal conditions, the most abundant terpenes determined were γ-E-bisabolene (WMW0052), caryophyllene (KMW0565), terpinolene (KMW0296), ocimene (KMW0252*154), bornyl acetate (WMW0016), β-pinene (KMW0193*119), m-cymene (XMW0126*016), and so on (**Supplementary Table 1**). It is obvious that the accumulation of most terpenoids examined was highest in MeJA-treated groups, which was similar to the change profiles observed in the relative odor activity value (**Figure 7**). Terpene synthases are capable of generating multiple terpenoid products with one substrate, thus largely resulting in numerous and different structures of plant terpenoids. Totally, 27 terpenoid synthase genes were found to be differentially expressed in response to MeJA application (**Figure 10**). Among them, 20 were distributed in clusters 2 and 3 during K-means analysis, corresponding to the change trends of most terpenoids.

### Co-expression of candidate terpenoid synthase genes and related transcription factors

**Figure 11:**
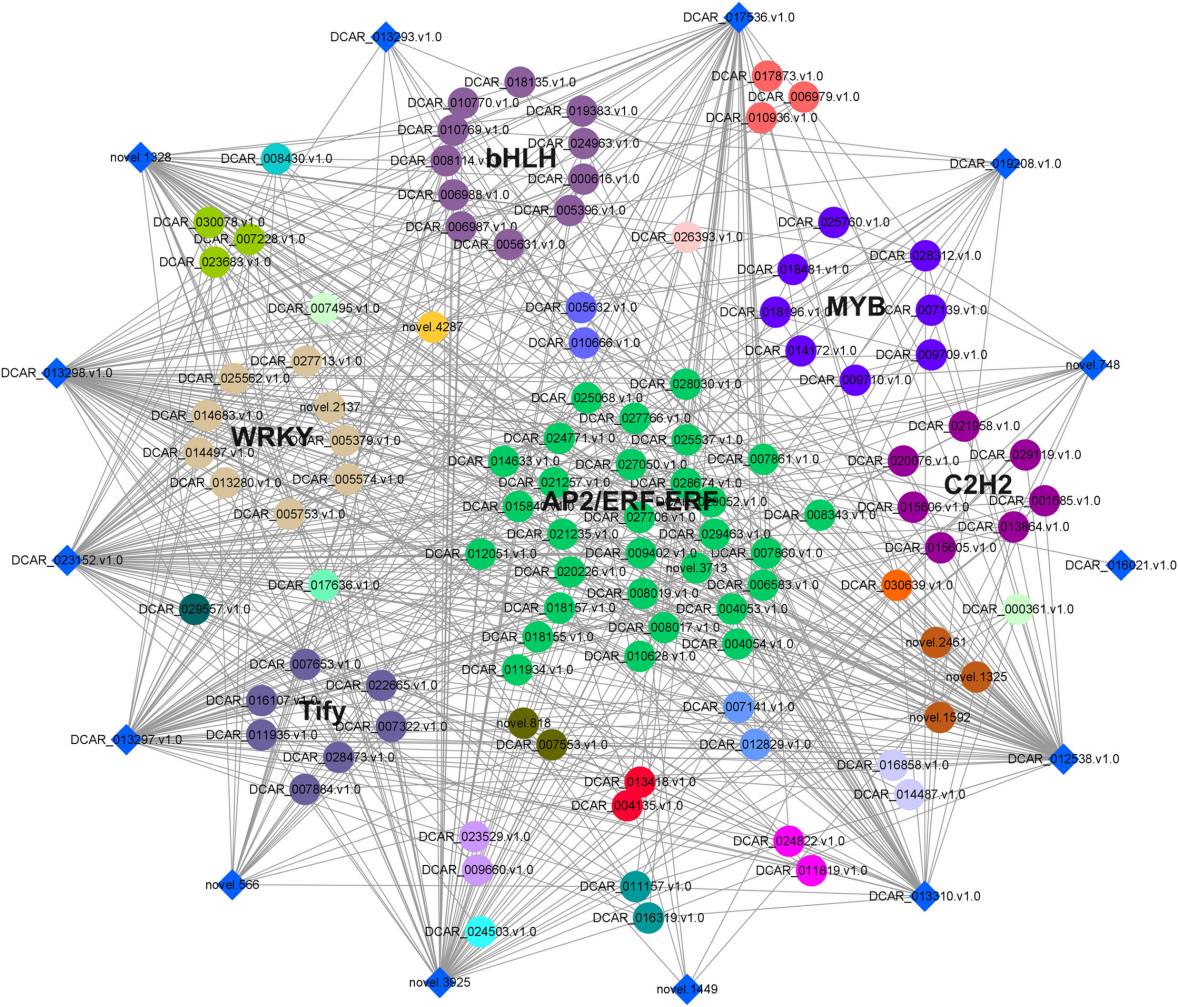
Co-expression network of TFs and terpenoid synthase genes in class 2 and 3 was constructed by Cytoscape (v.3.9.1) software. The diamonds around the periphery represented the structural genes, whereas different TF families were expressed as circles of different colors.

**Figure 12:**
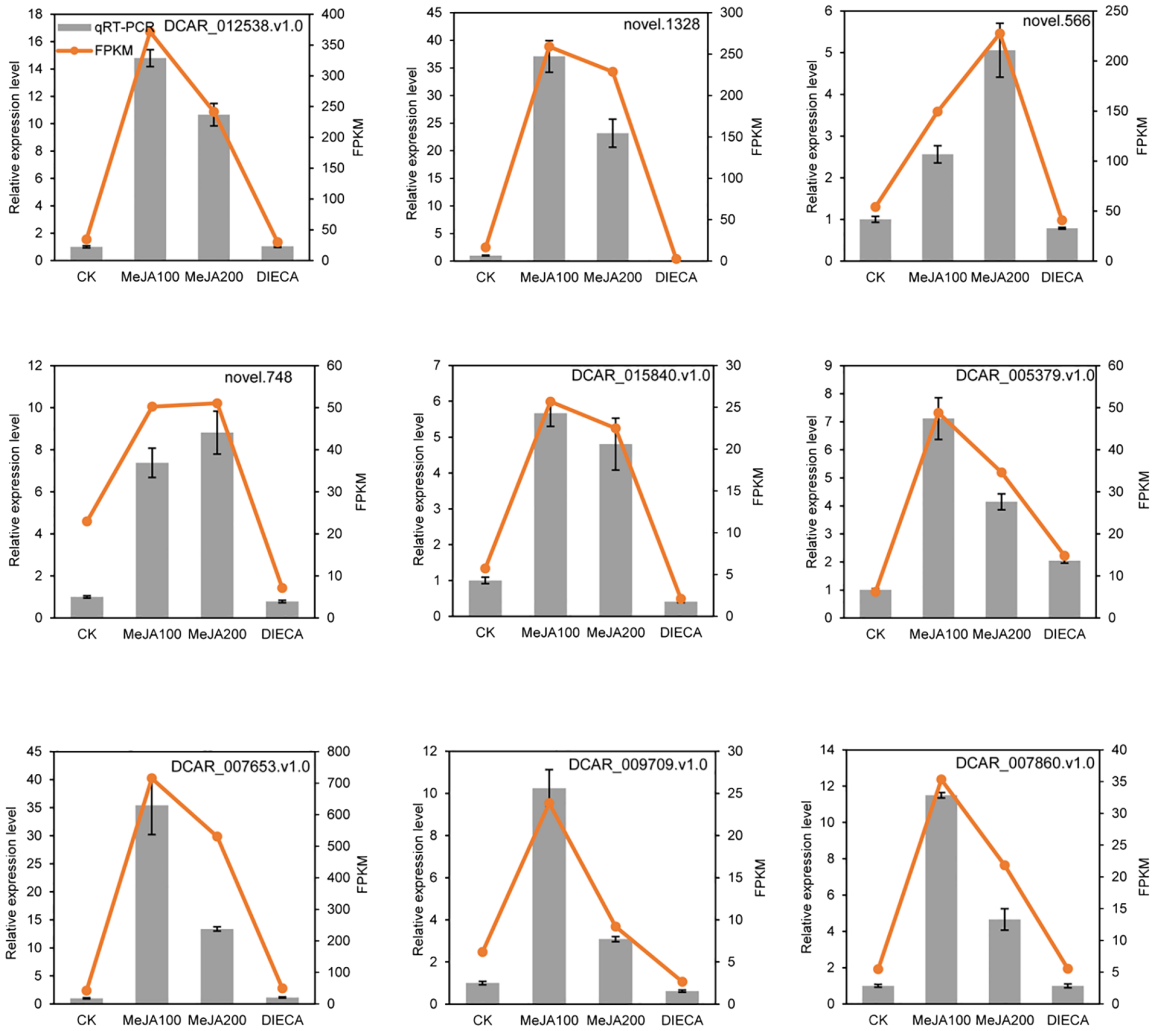
Quantitative real-time PCR validation of selected candidate genes. Error bars represent the standard deviation of three independent replicates.

To further examine the possible roles of transcription factors (TFs) in regulating flavor formation during MeJA application, the correlation between 20 structural (terpenoid synthase) genes and TFs within the same clusters was investigated based on fragments per kilobase of transcript per million mapped reads (FPKM) values (**Figure 11**). The results generated a complex network consisting of 14 structural genes and 106 TFs with 4, 680 TF-structural pairs that showed highly significant positive correlations (P ≤ 0.01, r ≥ 0.9). The most enriched TFs are ERF, bHLH, and WRKY, followed by MYB, C2H2, and Tify, indicating that these TFs might be involved in JA-mediated terpenoid accumulation indirectly by manipulating the transcript levels of structural genes. To confirm the accuracy of the digital gene expression from RNA-seq, 9 genes (4 structural genes and 5 TF genes) within the network were randomly selected for qRT-PCR analysis (**Figure 12**). The relative gene expression of all genes determined was well correlated with the RNA-seq data, suggesting a high credibility of the transcriptome results.

## Discussion

The aroma of horticultural products not only enriches people’s sensory experience, but may also have health benefits, and is an important component of the quality of horticultural products (Abbas et al., 2023). Horticultural products possess a special odor because of various volatile compounds within the plants, which play important roles in plant growth and development, defense mechanisms, and attracting pollinators (Hu et al., 2021; Picazo-Aragonés et al., 2020; Sharifi et al., 2022). Different horticultural products emit their own unique aromas due to their cultivars, growth environments, maturity, and processing methods (Chen et al., 2023; Karabulut et al., 2018). These special odors not only make horticultural products more attractive, but also give them unique flavors and nutritional value. Understanding and utilizing these odor characteristics in horticultural planting and food processing can create a more diverse range of products and experiences. Carrots are an important vegetable crop widely grown worldwide and loved by consumers (Coe et al., 2023). The characteristic volatile compounds in carrots are one of their main features and have been hot issues in quality breeding and scientific research in recent years. JA is an important regulatory factor in plant secondary metabolism, but can it regulate carrot flavor formation? What is its specific underlying mechanism? These issues have not yet been explained.

With the advancement and development of sequencing technology, people’s opportunities to obtain information on volatile metabolites have further been increased. In recent years, integrated transcriptome and metabolic analyses had contributed to elucidating the molecular mechanisms controlling VOC accumulation. In grape, bZIPs, AP2/ERFs, MYBs, and NACs were found to be implicated in the regulation of stress-responsive volatile metabolites by integrated network analyses (Savoi et al., 2017). Similarly, an integrated analysis of the volatilomics and transcriptomics revealed that the MYB-related transcription factor LATE ELONGATED HYPOCOTYL (JsLHY) may be involved in regulating the aroma formation of jasmine flowers, which was further demonstrated to activate the gene promoter regions of six aroma-related structural genes (Zhou et al., 2023). These results indicated that integrated multi-omics analysis had been an effective way to identify candidate genes responsible for volatile formation and regulation.

It is reported that terpenes account for approximately most of the flavor attributes in carrot, and much effort was devoted to investigating terpenoid accumulation and regulation (Keilwagen et al., 2017; Reichardt et al., 2020). In this manuscript, many other volatile compounds including esters, heterocyclic compounds, alcohols, ketones, hydrocarbons, and aromatics, were identified, although terpenoids still occupied the first place. It should be pointed out that some terpenes may also be classified as aromatic hydrocarbons or heterocyclic compounds according to different classification criteria for compounds. The components and relative contents of terpenes may be different owing to various carrot varieties, thus contributing to distinct aroma formation (Yahyaa et al., 2015). For example, a previous work indicated that the volatiles in the Kuroda type were relatively lower compared with those of other carrot types (Fukuda et al., 2013). Here, the terpenes present in great quantity detected in the control group were ocimene, γ-E-bisabolene, and bornyl acetate, followed by terpinolene, caryophyllene, β-pinene, and m-cymene (**Supplementary Table 1**).

JA may increase the odor intensity of horticultural commodities by altering different volatile compounds. Spraying MeJA pre-harvest can improve the concentrations of phenolics, thus conferring reinforced tolerance to chilling and decay during storage (Serna-Escolano et al., 2019). MeJA treatment promoted volatile accumulation and increased the contents of monoterpenoids and sesquiterpenoids in a dose-dependent manner (Dong et al., 2022). Similarly, in the current work, the accumulation of most volatile compounds was substantially increased after MeJA application, whereas DIECA did the opposite, suggesting a vital role of MeJA in VOC formation (**Figure 6**). JAs themselves are a type of derivatives of fatty acid with a unique aroma. Here, JA regulated the accumulation of terpenoids and the expression of terpenoid synthase genes. Most of the terpenoids and terpenoid synthase genes increased in response to JA, whereas some of them were inhibited, creating different terpenoid compositions and odor characteristics.

Plant transcription factors are a class of proteins that play a crucial role in the regulation of gene expression in plants. They regulate the transcription of genes by recognizing specific DNA sequences, thereby affecting plant growth, development, metabolism, and response to environmental changes (Han et al., 2023a; Kidokoro et al., 2022; Wang et al., 2023). Currently, Numerous studies have revealed that transcription factors are involved in the process of JA-mediated volatile metabolites. *MdMYC2* and *MdMYB85* from apple were strongly induced upon MeJA treatment and mediated the JA signaling to regulate ester aroma emission by collectively binding to the promoter of *ALCOHOL ACYLTRANSFERASE 1*, a gene responsible for ester synthesis (Li et al., 2023). Also, some transcription factors responsive to MeJA were also found to be correlated with terpene synthesis. *DobHLH4*, a transcription factor responsive to MeJA in *Dendrobium officinale*, directly interacted with the *DoTPS10* promoter and increased its expression, thus contributing to linalool biosynthesis (Yu et al., 2021). Similarly, a MeJA-induced bHLH transcription factor *TaMYC2* from dandelion, could act as a positive regulator of triterpene accumulation by binding to the promoter of the *squalene synthase* (*TaSS*) gene (Liu et al., 2023). Although several terpenoid synthases that can manipulate terpenoid metabolism have been identified in carrot, there is limited research on its transcriptional regulation. So far, no transcription factor has been reported to regulate carrot terpenoid biosynthesis. Here, 4,680 TF-structural pairs were identified after MeJA spraying, which can serve as positive gene resources for in-depth research on the regulation of terpenoid accumulation, thus providing precise targets for enriching carrot flavor quality.

To our knowledge, this is the first work of global volatile metabolome analysis and characterization of differential accumulated metabolites in carrot upon MeJA treatment. The results generated 1,227 volatile organic compounds and 972 differential accumulated metabolites, with terpenes accounting for the largest group. MeJA treatment strongly increased the relative odor activity values as well as the accumulation of most volatile compounds. In addition, 4,787 differentially expressed genes were further identified to understand the potential underlying mechanism. The expression profiles of terpenoid synthase genes and their correlations with transcription factors were extensively investigated. This study improved our understanding of the methyl jasmonate-mediated responses in carrot at the metabolic and molecular levels.

## Data Availability

The datasets presented in this study can be found in online repositories. The names of the repository/repositories and accession number(s) can be found in the article/supplementary material.
